# Mechanism of COVID-19-Induced Cardiac Damage from Patient, In Vitro and Animal Studies

**DOI:** 10.1007/s11897-023-00618-w

**Published:** 2023-08-01

**Authors:** Elizabeth A. V. Jones

**Affiliations:** 1 Centre for Molecular and Vascular Biology, Herestraat 49, Bus 911, 3000 KU Leuven, Belgium; 2 Department of Cardiology, CARIM School for Cardiovascular Diseases, Maastricht University, Universiteitssingel 50, 6229 ER Maastricht, Netherlands

**Keywords:** COVID, Microthrombi, Pericyte, Heart

## Abstract

**Purpose of Review:**

Though patient studies have been important for understanding the disease, research done in animals and cell culture complement our knowledge from patient data and provide insight into the mechanism of the disease. Understanding how COVID causes damage to the heart is essential to understanding possible long-term consequences.

**Recent Findings:**

COVID-19 is primarily a disease that attacks the lungs; however, it is known to have important consequences in many other tissues including the heart. Though myocarditis does occur in some patients, for most cases of cardiac damage, the injury arises from scarring either due to myocardial infarction or micro-infarction.

**Summary:**

The main focus is on how COVID affects blood flow through the coronaries. We review how endothelial activation leads to a hypercoagulative state in COVID-19. We also emphasize the effects that the cytokine storm can directly have on the regulation of coronary blood flow. Since the main two cell types that can be infected in the heart are pericytes and cardiomyocytes, we further describe the known effects on pericyte function and how that can further lead to microinfarcts within the heart. Though many of these effects are systemic, this review focuses on the consequences on cardiac tissue of this dysregulation and the role that it has in the formation of myocardial scarring.

## Introduction

Chest pains remain one of the main complaints that lead COVID-19 patients to the hospital [1] and throughout the pandemic, there have been fears regarding possible long-term effects to the heart. Cardiovascular disease and hypertension are important comorbidities that affect the severity of COVID-19 [2]. ACE2, the receptor used by SARS-CoV-2 to enter cells, is more highly expressed in the myocardium than in the lungs, even though COVID-19 primarily affects the lungs [3]. Thus, there were significant concerns of major cardiac involvement early in the pandemic. While many of those fears have since been allayed, we have a better understanding of what is happening in the heart during SARS-CoV-2 infection and the possible long-term consequence this infection has. In this review, we focus on what is known about damage to the heart that can occur in some cases and the evidence of the molecular/physiological mechanisms of this damage.

Initial reports had created concerns because there were reported incidences of myocarditis between 2%–15% even in young athletes [4]. As numbers increased, however, it became clear that true cardiac involvement was relatively rare in young athletes [5]. Even in those that had shown cardiac involvement, the myocarditis was resolved at a one-year follow-up [6]. In hospitalized patients, however, some patients did present with myocarditis, where 2.4 per 1000 hospitalizations were diagnosed as definite/probable myocarditis and an additional 4.1 per 1000 were considered as possible myocarditis.

Low rates of myocarditis may reflect the relatively short period of time that the virus remains in the heart for most individuals. Work in animal models is largely limited to hamsters, that are naturally susceptible to SARS-CoV-2, or transgenic mice that have forced expression of human ACE2. In the hamster, viral transcripts and both the spike and capsid protein can be found in cardiomyocytes of the left and right atrium two days post infection, and the left ventricle but surprisingly not the right ventricle [7]. Viral RNA is present at relatively high levels 4 days post infection, but is essentially undetectable by 14 days, a time at which viral RNA is still detectable at high levels in the lung [8]. Hamster’s show weight loss starting 2 days after infection [9], and as such, the point where virus is detectable in cardiomyocytes may only be for a short period after the initial infection. In vitro, iPSC-derived cardiomyocytes show a strong ability to be infected, but cells were only followed for at most 5 days post infection [7, 10]. In sections of hamster hearts, cardiomyocytes were not overtly positive for SARS-CoV-2 viral mRNA by in situ hybridization by 4 days post infection [8]. Similarly, post-mortem cardiac tissues identify spike protein and nucleoprotein on sections, but it is a minority of cardiomyocytes that stain positive [11]. Thus, though the virus can infect cardiomyocytes, the infections is likely short-lived. This could explain why various groups have reported that they either fail to find any viral RNA in the heart or find only very low levels of virus [12–14]. The point of autopsy is just too long after initial infection and the active virus has cleared, even though the systemic hypercoagulatory state is still present.

Even though only a minority of COVID-19 patients experience myocarditis, this does discount possible serious cardiac consequences to having the disease. Markers of cardiac damage such as NT-proBNP, cTn and d-dimer, can be elevated in COVID-19 patients and correlate to disease severity [15, 16]. Between 8–62% of hospitalized patients with COVID-19 show elevated troponin levels [15, 17–19]. Increased troponin associates with increased disease severity and likelihood of dying [15, 19]. The increased troponin levels also correlate to damage visible by CMR. In the COVID-HEART study, 342 hospitalized patients with COVID-19 that had presented with high troponin levels were imaged. Of those patients, 61% showed signs of myocardial injury including left or right ventricular systolic dysfunction, myocardial scar, or pericardial effusion [20]. Furthermore, the scar pattern was different than patients with myocardial scarring from other diseases. Myocarditis was suggested in 1 of 20 cases, but the vast majority showed signs of myocardial infarction and microinfarction [20]. The amount of myocardial scarring, and not initial troponin levels, were predictive of future major adverse cardiovascular events [20].

If scarring is the most important cardiac consequences with respect to future possible events, it is important to understand why scarring of the heart occurs and what is inducing these microinfarctions. The reason for the damage is several-fold. First, endothelial cell activation leads to microthrombi formation (also called disseminated intravascular coagulation or DIC). Larger thrombi can directly cause a myocardial infarct, but the microthrombi cause flow disturbances. Second, pericyte death and dysfunction further exacerbates flow abnormalities. Lastly, the cytokine storm affects endothelial dependent and independent vasoconstriction/dilation further perturbing flow patterns. These mechanisms all work together to create a failure of microcirculatory flow in the heart, leading to regionalized and possibly transitory hypoxia that create the microinfarct. We would further suggest that there is an exaggerated ischemia–reperfusion reaction, though this step is speculative. We will therefore explore each of these events individually, with a focus on the heart.

## Endothelial Cell Damage and Microthrombi Formation

Endothelial cell damage and activation promotes coagulation in COVID-19 leading to both macro and microthrombi formation [21]. Though large occlusions can form with COVID-19, most thrombi are smaller and do not completely occlude the coronary vessels. Examining the hearts of 40 patients that died from COVID-19, 35% had signs of myocyte necrosis but only 21% of those showed signs of an acute myocardial infarction whereas 79% had focal myocyte necrosis [22]. Using a hamster model where perfusion can be directly assessed by injection of a labelled tomato-lectin, our group found a large increase in fibrin-rich thrombi, no change in vWF-rich thrombi, but also no effect on the labelling of perfused vessels [8]. The injected tomato-lectin labels only the blood plasma, however, and does not indicate whether the circulation of red blood cell is perturbed. Any decrease in diameter is likely to impede red blood cell circulation but not necessarily the flow of blood plasma. The results from the animal model mirror what has been found on autopsy. The lungs of deceased COVID-19 patients show thrombi composed of platelets and fibrin [21, 23]. In the heart, microthrombi were also fibrin rich and did not completely occlude vessels [12, 22].

The vasculature has an active role in preventing coagulation at rest. Endothelial cells express tissue factor pathway inhibitors (TFPI), that blocks the initiation of coagulation [24]. Endothelial cells also produce heparan sulfate, a vital part of the glycocalyx layer than lines all blood vessels. Heparan sulfate inhibits binds antithrombin, resulting in a 2000-to-4000-fold increase in its activity [25]. Antithrombin binds thrombin preventing its pro-coagulant activity [26]. Interestingly, the spike protein of SARS-CoV-2 binds heparan sulfate at high affinity [27], which may competitively block heparan sulfate from preventing coagulation. Blood from patients with COVID-19 also shows elevated heparinase activity and could induce shedding of heparan sulfate from endothelial cells exposed in vitro for 30 min to plasma from patients [28]. Endothelial cells produce thrombomodulin which binds thrombin and converts thrombin from pro-coagulant to anti-coagulant [26, 29]. Endothelial cells also produce nitric oxide that prevents platelet aggregation [30]. Hospitalized COVID-19 patients had lower arginine levels, which may indicate that NO bioavailability is also reduced [31]. When endothelial cells are treated with plasma from COVID-19 patients for 30 min, they showed significantly lower levels of nitric oxide production [28]. Lastly, healthy endothelial cells are essential to prevent coagulation since endothelial cells sequester vWF, p-selectin and chemokines in Weibel Palade Bodies (WPB). Levels of vWF factor are severely elevated in COVID-19 patients [32, 33], even in hospitalized patients with relatively moderate symptoms [34]. Incubating endothelial cells with plasma from ICU COVID-19 patients for 10 min induced WPB exocytosis and secretion of vWF [34]. As such, there is elevated hypercoagulative state present upon infection.

There are three necessary components to thrombus formation: damage to the vessel wall, alterations in blood flow and changes to blood composition. This is referred to as Virchow’s triad [35]. Endothelial cell damage has been recognized as central to disease progression, and yet it is highly debated whether endothelial cells themselves can be infected to any significant extent. Viral particles have been reported in endothelial cells as imaged by TEM [21, 36], however the identification of the virus in these images have been questioned [37–39]. And whether endothelial cells even express ACE2 is highly controversial. The early papers on the discovery of protein ACE2 all reported endothelial expression. However, many of those did so by immunostaining or in situ hybridization and without co-staining for endothelial markers [40–42]. And while the question of whether endothelial cell express ACE2 should easily be resolved by single cell RNA-Seq experiments, even those results are contradicting, with some results indicating little or no expression [43–46] and other stating that there is high expression in endothelial cells [47]. In papers that specifically looked at the heart, only cardiomyocytes and pericytes were found to have any significant expression of ACE2 [46, 48, 49]. It may, however, be that virus can only enter endothelial cells under certain disease state or that COVID-19 amplifies expression of ACE2 in endothelial cells allowing viral entry [50]. But even if the virus can enter endothelial cells, multiple groups have found that the virus cannot proliferate in endothelial cells [45, 51, 52]. As such, the concentration of virus in endothelial cells would be low compared to other cell types such as pericytes and cardiomyocytes.

Though the mechanism by which the endothelium becomes damaged is debated, there is absolutely no disagreement in the field that endothelial cells are damaged by COVID-19. Early histology from patients who had died from COVID-19 shows “plump” endothelial cells [53]. SEM images of the corrosion cases of the lung vasculature from a patient who died of COVID-19 show tortuous and distorted capillaries in the lung [21]. Exposing endothelial cells to plasma from COVID-19 patients for 1 h causes decreased viability and increased secretion of vWF [54]. Given the short exposure time, this cannot be due to direct infection which takes much longer. Furthermore, increased levels of circulating endothelial cells are detected in COVID-19 patients and higher levels of circulating endothelial cells correlated with disease severity [55, 56]. Most circulating endothelial represents unhealthy cells that were sluffed off. Not only does denudation of the blood vessel induce coagulation, but there is also evidence that endothelial cells are senescent in COVID-19 [57]. Senescent endothelial cells were shown to upregulate coagulation factors more strongly in response to SARS-CoV-2 than non-senescent cells [58].

## Direct Effects of the Cytokine Storm on Cardiac Perfusion and Function

Focal ischemia may also occur, at least in part, due to direct effects of cytokine storm on coronary vascular function. Damage to the lung parenchyma causes the release of damage-associated molecular patterns (DAMPs) that activate the innate immune cells to produced factors such as TNF-α [59]. TNF-α increases the rate of eNOS mRNA degradation leading to reduced endothelial dependent relaxation [60]. TNF-α not only decreases NO production, but it also increases microvascular tone in arterioles in an endothelial-independent manner [61, 62]. As mentioned, not only is eNOS mRNA reduced but arginine levels, needed to produce nitric oxide, are significantly lower in COVID-19 patients. As such, not only are there microthrombi dysregulating flow patterns, but vasoreactivity is also completely dysregulated. In the lung, ARDS associated inflammatory processes are known to trigger vasodilation in non-ventilated areas and vasoconstriction in ventilated areas [63]. This is largely driven by dysregulation of nitric oxide signaling by cytokine storm that is likely to be present in all tissues, including the heart.

Dysfunction in endothelium-dependent vasoregulation in COVID-19 patients is supported by measurement of flow-mediated dilation (FMD). In young adults who had experienced mild to moderate symptoms, FMD was significantly reduced 3–4 weeks after initial infection [64]. Though this improved in the months following infection, it remained lower than control measurements even six months after infection [65]. Impaired flow mediated dilation persists in patients that had severe COVID-19 even one year after initial infection [66]. These changes are important since decreases in FMD alone are associated with increased risk of heart attack, stroke, or death [67].

Nitric oxide is not the only flow-regulating molecule that is dysregulated by the cytokine storm. Most of the anti-coagulant pathways that are affected in COVID-19 also act as potent vasodilators, such as prostacyclin. Vasoconstrictors such as Endothelin-1, on the other hand promote coagulation [68]. IL-6 causes upregulation of angiotensin II type 1 receptor, causing increased angiotensin II-mediated vasoconstriction of smooth muscle cells [69]. As such, there is a complete dysregulation of normal microvascular flow patterns with a combination of increased and decreased contractility.

Capillary leak syndrome is a classic component of a cytokine storm. Capillary leak syndrome refers to the increase in permeability that occurs during a cytokine storm, and it leads to hypotension, edema, acute respiratory failure, and kidney injury [70]. In patients that died from COVID-19, a breakdown of the blood brain barrier was observed in more than 50% of patients, as assessed by staining for extravascular fibrinogen [71, 72]. Cytokine storm are known to induce increase vascular permeability because of the presence of VEGF in the serum that leads to internalization of VE-Cadherin and thereby disassembly of the adherens junctions [73]. Circulating IL6 can also increase vascular permeability. IL6 binds a soluble form of IL6R (called sIL-6R) that is present in blood plasma and exposing endothelial cells to this complex results in internationalization of VE-Cadherin of the adherens junctions and increased permeability [74]. The cardiac consequence of capillary leak syndrome is cardiac edema. In a hamster model, SARS-CoV-2 infection led to an immediate increase in water content in the heart showing that edema happens very rapidly after initial infection, present at the earliest stage investigated, 4 days post infection [8]. This increased water content resulted in almost immediate swelling of the cardiomyocytes after infection [8]. Cardiac edema secondary to cytokine storm has been previously reported [75–77]. Reports consistently show diffuse myocardial edema in COVID-19 patients [78–82], that for the most part resolves within approximately 6 months [83]. Similar to the results in the animal model, this was reported as an intra-cardiomyocyte edema [81]. Edema is also classic symptom of myocarditis, however the link between patients that present with edema and those that have myocarditis has currently not been made. Cardiac edema reduces ventricular compliance and affects both systolic and diastolic function [84].

And while the cytokine storm can affect nutrient and oxygen delivery to the heart through its control on proper circulation, the high levels of cytokines also have direct effects on cardiomyocyte function. TNFα, interleukin-6, and interleukin-2 have all been shown to inhibit cardiac muscle contraction in a concentration-dependent reversible manner [85]. Il1β treated cardiac organoids produce fibrosis and display reduced contractility [86]. The cytokine storm of COVID-19 products cytokines that are very similar in profile to the surge of cytokines produced during septic cardiomyopathies [87]. Except for the fibrosis, most of these effects, however, should be reversible after patient recover.

## Pericyte Dysfunction

In the heart, the two cell types with the highest expression of ACE2 are cardiomyocytes and pericytes [3]. Various groups have shown that pericytes can be infected in the heart [8, 14, 88]. In a hamster model, infection leads to a loss in pericytes coverage on capillary vessels that takes over a month to recover to pre-infection levels [8]. Many of the comorbidities associated with aggravated reaction to SARS-CoV-2 infection, such as diabetes and hypertension, are associated with altered pericyte function [89–92]. Therefore, it is easy to speculate that pericyte recovery would be either be more altered or delayed in these patients. This function of this cell type is poorly understood and therefore the functional consequences on the heart are not clear.

Pericytes have many roles in microvascular physiology but one of the main roles is to keep endothelial cells quiescent. In a genetic model of pericyte loss, endothelial cells were shown to significant upregulate vWF mRNA levels [93], indicating that pericyte loss may play a role in the hypercoagulable state after SARS-CoV-2 infection. Loss of pericytes directly affects endothelial cells. The PDGF-B^ret/ret^ mice, which show an 80% reduction in capillary coverage, exhibit increased VCAM-1 and ICAM-1 expression even in the absence of any pro-inflammatory signals [94]. Pericytes are also important mediators of inflammatory signals. Pericytes create a second checkpoint for leukocyte migration out of the vasculature. After migrating through the endothelial layer, leukocytes migrate in the subendothelial space between endothelial cells and pericytes until they find a permissive region to extravasate [95]. The gaps between pericytes that are permissive an enlarged during inflammation [96]. Reduced pericyte coverage therefore leads to large increases in leukocytes infiltration [94]. Furthermore, pericyte express receptors for cytokines including TNFRI, TNFRII, and IL-1RI [96], indicating that they too are responsive to increased circulating cytokines that occurs in COVID-19. In response to inflammatory signals including LPS but also DAMPs, pericytes secrete factors that induce chemotaxis of myeloid leukocytes [97]. In the CNS, various groups have also reported that pericytes modulate T-cell infiltration [98–100]. Altogether, it is safe to assume that the loss of pericytes in the heart exaggerates the immune response to SARS-CoV-2 infection.

Pericyte death can have profound effects on blood flow patterns in the microcirculation and therefore the loss of pericyte is likely to exacerbate the circulatory defects caused by the microthrombi. In the brain, it has been found that pericyte can either detach and migrate away from vessels [101, 102], or they can die and remain in a state of rigor mortis [103]. The latter is thought to occurs mainly in hypoxic environments. Optical ablation of single pericytes in vivo leads to increased flow through the capillary vessel in question [104]. As such, loss of pericyte coverage would lead to increased flow in regions. Death in a state of rigor mortis has been shown to constrict vessels and reduce flow through that vessel. Interestingly, binding of the virus to ACE2 may also affect pericyte function. In a hamster brain slice culture system, exposing pericytes to Angiotensin II leads to about a 10% reduction in capillary diameter over 2.5 min. In the presence of the receptor binding domain of SARS-CoV-2, the response is exaggerated, with a 40% reduction in capillary diameter over the same time period [105]. The combination of all these factors results in a microvasculature consisting of regional capillary vasodilation that effectively creates microvascular shunts, and other pathways that have largely increased resistances to flow. In dementia, the combined loss and increase in capillary resistance has been proposed to lead to regional hypo-oxygenation due to increased heterogeneity of capillary transit time [106]. As blood flows too quickly through some regions, it fails to properly oxygen that region. In other regions with increased resistance to blood flow, too little blood flows and it is also improperly oxygenated. Though SARS-CoV-2 infection has been shown to lead to rapid pericyte loss [8], there current is no evidence that it can also lead to a state of rigor mortis. If pericyte loss is secondary to hypoxia, however, a state of rigor would be likely [103]. Even in the absence of rigor mortis, the presence of microthrombi could play a similar physiological role by increasing the resistance to flow in some vessels. As such, COVID-19 has been proposed to alter capillary transit paths leading to hypoxic conditions [107].

## Ischemia–Reperfusion Injury

Viruses uses a range of tactics to counter the host’s antiviral responses. All coronary viruses, including SARS-CoV, MERS-CoV and SARS-CoV-2, are known to shut down transcription of host RNA in favor of the transcription of viral RNA [108]. Beyond shutting down the host’s transcription, coronaviruses also inhibit specific transcription factors involved in the immune response. SARS-CoV and SARS-CoV-2 inhibit activation of interferon signaling, and the extent of interferon response suppression has been proposed as a determinant of disease severity [109]. The ORF6 protein of SARS-CoV can inhibit STAT1 translocation [110], an important step in the interferon response. It is unclear if SARS-CoV-2 has the same ability. Infection of Calu-3 cells with SARS-CoV-2 did not prevent IRF3 phosphorylation, nor STAT1 or P65 translocation as it did with infections with SARS-CoV-2 [111]. Other groups similarly found that overexpression of ORF proteins from SARS-CoV-2 only slightly inhibited p65 translocation (between 70 and 85% of normal translocation after TNF-α induction) [112]. However, a third group found that SARS-CoV-2 infection indeed interfered with STAT1 translocation in Vero E6 cells [113].

The reason that this interference is interesting to cardiac research is that ORF6 has been proposed to inhibit translocation by interacting with nuclear pore proteins. Mutations in nuclear pore proteins affect cardiac function, as most clearly shown by mutations in Lamin A/C that induce dilated cardiomyopathy [114]. In COVID-19, the ORF6 directly interacts with nuclear pore protein Nup93, as indicating by co-immunoprecipitation [113]. This could indicate that after infection, Nup93 function is inhibited. Knockdown of Nup93 in neonatal cardiomyocytes results in an almost 4 × increased expression of ANP and BNP [115]. BCL2 induction by hypoxia was inhibited with knockdown of Nup93 in neonatal cardiomyocytes. Furthermore, this knockdown aggravates hypoxia induced injury of cardiomyocytes [115].

Our own results showed the HIF-1α translocation was inhibited in the cells where SARS-CoV-2 is present. HIF-1α binds to the ACE promoter and results in increased ACE expression and decreased ACE2 expression [116, 117]. It therefore would be beneficial for the virus to block its function. Functionally, however, our results are difficult to resolve with other published results. Other research groups have looked at HIF1α signalling, but most only report an increase in either RNA or protein which does not indicate that the transcription factor is reaching its target [118, 119]. In fact, overexpression of SARS-CoV-2’s ORF3a and ORF6 causes increased HIF1a mRNA levels [119] which could arise because of feedback where the genes are not being upregulated even though HIF-1α is expressed. One group did stain for HIF1α in patient samples, however the staining was limited to endothelial cells, which as previously discussed, may not be infected with SARS-CoV-2 [120]. In disagreement, however, HIF1α targets have been shown to be upregulated in lungs from COVID-19 patients [121]. It was also showed that HIF1α targets such as IFN-beta, IL6, IL1beta, are upregulated by SARS-CoV-2 in Caco-2 cells, and this upregulation is prevented by a HIF-1α inhibitor [119]. This would therefore indicate a functional consequence to the HIF-1α expression.

If normal response to hypoxia is inhibited, whether through decreased HIF-1α translocation (as we saw) or generally through Nup93 inhibition (as other saw), then we would expect an exaggerated ischemia–reperfusion response. Mice heterozygous for HIF-1α show increased severity of ischemia–reperfusion injury [122, 123]. The presence of virus is, however, short-lived in the heart, as explained above. Therefore, normal HIF-1α should return as the virus is cleared of the heart within days of infection.

## Conclusion

On May 5, 2023, the WHO announced the end of the COVID-19 public health emergency [124]. As hospitalization becomes less common after infection, the current concern regards long term consequences of infection. In the heart, the greatest concerns relate to patients showing myocardial scarring [20]. Understanding the origin of this scarring can help us understand future possible consequences on the heart. Furthermore, given that this is the third coronavirus outbreak in recent years, it is important to understand the mode of action of this type of virus for possible future outbreaks. The overall mechanism appears to be driven by flow disturbances and inadequate regional perfusion leading to microinfarctions, and sometimes macro, throughout the heart.
